# Factors Associated with Successful Treatment by Radiofrequency Treatment of the Soft Palate in Obstructive Sleep Apnea as the First-Line Treatment

**DOI:** 10.1155/2015/690425

**Published:** 2015-08-03

**Authors:** Nuntigar Sonsuwan, Kongsak Rujimethabhas, Kittisak Sawanyawisuth

**Affiliations:** ^1^Otolaryngology Department, Faculty of Medicine, Chiang Mai University, Chiang Mai 50200, Thailand; ^2^Chiangrai Prachanukroh Hospital, Chiang Rai 57000, Thailand; ^3^Department of Medicine, Faculty of Medicine, Khon Kaen University, Khon Kaen 40002, Thailand; ^4^Research Center in Back, Neck Other Joint Pain and Human Performance (BNOJPH), Khon Kaen University, Khon Kaen 40002, Thailand

## Abstract

*Background*. Radiofrequency ablation (RFA) is recommended as the second-line treatment for obstructive sleep apnea (OSA). This study aims to study the factors associated with successful treatment by RFA in OSA patients as the first-line treatment. *Methods*. All patients diagnosed as mild to moderate OSA were enrolled prospectively and treated with RFA. Three points of soft palate were ablated: midline, left, and right paramedian sites. Baseline characteristics and clinical factors including snoring score (SS), Epworth Sleepiness Scale (ESS), and apnea-hypopnea index (AHI), with minimal oxygen saturation, were recorded at baseline and three months after treatment. *Results*. During the study period, there were 51 patients who met the study criteria and received RFA treatment. At three months, the SS, ESS, and AHI were significantly lower than baseline values, while the minimal oxygenation was significantly increased from the baseline values. There were 16 patients (31.37%) who had an AHI of < 5 times/h. Only baseline AHI was significantly associated with an AHI of < 5 times/h at three months after RFA treatment. The adjusted odds ratio was 0.804 (95% CI: 0.699, 0.924). *Conclusion*. Transoral RFA treatment may be effective in mild to moderate OSA as the first-line treatment. Baseline AHI is associated with preferable outcome by RFA treatment.

## 1. Background

Obstructive sleep apnea (OSA) is a common disease in general practice. Its prevalence is approximately 5–10% of the general population [1]. If left untreated, cardiovascular consequences may occur such as stroke, cardiac arrhythmia, or hypertension [2]. Continuous positive airway pressure (CPAP) machines have been shown to be the most effective treatment [3]. Major cardiovascular events and resistant hypertension are under control with the CPAP therapy [4–6]. Only half of OSA patients are able to comply with the CPAP treatment. Several treatment modalities have been studied to be alternative choices for OSA patients such as an oral appliance or a surgical procedure.

Radiofrequency ablation (RFA) treatment is effective to reduce snoring and also sleepiness symptoms [7]. The procedure can be performed in the ambulatory setting and needs only local anesthesia. RFA is recommended as the second line treatment for mild to moderate OSA if CPAP therapy is not adhered to or tolerated [8]. Data regarding OSA and RFA treatments in Thai or oriental patients are limited [9]. OSA patients in an Asian population may be different from the Western world due to different anatomical features and genetic factors [10]. Here, the effect of RFA in mild to moderate OSA Thai patients was evaluated. In addition, factors associated with successful treatment by transoral monopolar RFA treatment in obstructive sleep apnea as the first-line treatment were studied.

## 2. Methods

The study was conducted at the Department of Otolaryngology, Chiang Mai University, from October 1st, 2007, to September 30th, 2008. All patients who presented with snoring and were diagnosed as mild to moderate OSA (AHI 5–29 times/hour) were enrolled prospectively. The diagnosis of OSA was made using Somnocheck V2.04. The patients were invited to participate in the study and receive RFA as the first-line treatment for the OSA. The study protocol was approved by the Research Ethics committee, Chiang Mai University. Before the enrollment, the eligible patients were introduced to the study protocol and signed the informed consent.

Patients were excluded if one of the following conditions existed: body mass index of more than 30 kg/m^2^, presence of nasal or supraglottic obstruction, coexisting sleep disorders such as insomnia, periodic limb movement disorders, pregnancy or pregnancy plan, and comorbid diseases that may affect wound healing processes such as diabetes, cardiovascular diseases, coagulopathy, and incomplete clinical data.

RFA was performed by an ENT physician. Transoral monopolar radiofrequency treatment with an Ellman device (Ellman Surgitron model EMC Ellman International, Inc., Ocean side, New York) was used. The frequency used was 3.8 MHz in the coagulation mode with power at 3.5 which produced a current of 58 Watts resulting in a temperature of 69-degree Celsius which was performed with local anesthesia. The bendable 2 cm long needle electrode was inserted into the midline soft palate at the level about 5 mm below soft and hard palate junction. The other two ablation sites were at left and right paramedian area, 7-8 mm laterally from midline [11]. These procedures were performed with the active and insulator needle electrodes.

Clinical data were collected at baseline and three months after RFA treatment including baseline characteristics, snoring score (SS), Epworth Sleepiness Scale (ESS), and AHI. The pain score and ulcer occurrence after RFA treatment were monitored at one week after RFA. The pain score was scored by patients with the maximum pain at 10. The SS was defined by patients and/or bed partners with range of 0–10 in the visual analogue scale. The scores were rated by loudness of snoring and effects on the bed partners: 0 indicated no snoring, and 10 indicated very loud snoring with bed partners or others having to leave the bedroom. Patients were classified into two groups by the AHI at three months if less than 5 times/hour or more.

### 2.1. Statistical Analyses

Baseline and clinical characteristics of patients with AHI at three months less than 5 times/hour versus equal to or more than 5 times/hour were compared using descriptive statistics. Wilcoxon rank-sum and Fisher's exact tests were applied to compare the differences in numbers and proportions between the two groups. Pre- and post-SS, ESS, AHI, and minimal saturations were compared between the groups by the paired *t*-test.

Univariate logistic regression analyses were applied to calculate the crude odds ratios of individual variables for AHI less than 5 times/hour. All clinical variables were considered statistically significant if the *p* value by univariate analysis was less than 0.20 and were included in subsequent multivariate logistic regression analyses. Factors that had collinearity with other factors were not included in the model. Analytical results were presented as crude odds ratios (OR), adjusted OR, and 95% confidence intervals (CI). All data analyses were performed with STATA software (College Station, Texas, USA).

## 3. Results

During the study period, there were 62 patients who were eligible and interested to participate in the study. Six patients were excluded due to a baseline AHI of less than 5 times/hour, while 5 patients had an AHI 30 times/hour. In total, 51 patients completed the study protocol. At three months, the SS, ESS, and AHI were significantly lower than the baseline values (Table 1), while the minimal oxygenation was significantly increased from the baseline values. There were 16 patients (31.37%) who had an AHI less than 5 times/hour.

Clinical features between those who had an AHI at three months less than 5 times/hour versus 5 times/hour or more are shown on Table 2. Most factors were comparable between the groups except baseline AHI, posttreatment AHI, baseline minimal saturations, and posttreatment minimal saturations. The median baselines of the AHI of patients who had an AHI less than 5 times/hour versus those equal to or more than 5 times/hour at three months were 8 and 17 times/hour. After being adjusted for variables shown in Table 3, only baseline AHI was significantly associated with an AHI less than 5 times/hour at three months after RFA. The adjusted odds ratio was 0.804 (95% CI: 0.699, 0.924).

There were three patients who had self-limited mucosal ulcer after RFA: size 1, 3, and 5 mm. The median pain score at one week of treatment was 0 and 0.5 in patients who had the AHI equal or more than 5 times/hour and less than 5 times/hour at three months after treatment.

## 4. Discussion

The present study showed that RFA is beneficial for mild to moderate OSA as a first-line treatment with very few complications. The main mechanism for RFA in OSA is to enlarge airways by shrinking and stiffening of the tissue [7].

The successful treatment of OSA is defined by having a posttreatment AHI less than 5 times/hour [6]. The successful rate of RFA in the present study was 31.37% which was comparable with previous studies [12, 13]. Riley et al. found that RFA successfully treated OSA patients at 32.4%, while Stuck et al. had the success rate of RFA of 33%. Patients with an AHI less than 15 times/hour and a body mass index less than 23 kg/m^2^ had a higher chance for successful RFA [12]. In this present study, the median AHI and body mass indices of patients who had successful treatment were 25.20 kg/m^2^ (range 22.50–29.00) and 8 times/hour (range 5–25). After adjustment for confounding factors, only baseline AHI was associated with successful RFA with an adjusted OR of 0.804. Increasing the time of 1 time/hour of AHI reduces the chance of successful RFA by 20%.

In addition, RFA improved all other outcomes including SS, ESS, and minimal saturation. These results were compatible with previous studies [11, 12, 14, 15]. Other than sleepiness symptoms and snoring score, RFA was shown to improve patients' quality of life as well [8]. The advantage of RFA is that the procedure is performed only one time and its effect may last for at least almost two years for OSA symptoms, quality of life, and sleep parameters [16].

The strengths of this present study were prospective data collection and the study population. The results confirm that RFA may provide successful treatment in mild to moderate OSA as a first-line treatment. Even though the follow-up period of this study was quite short at three months, previous studies showed that the effect of RFA may last up to 26 months [16, 17]. For those who still had a high AHI at three months, repeated RFA may be another treatment option due to the low complications of RFA. Only three patients developed soft palate mucosal ulcers which were small and self-limited. Post-RFA pain disappeared after one week in almost all patients [14, 18]. However, the reduction of AHI may be partly due to the regression to the mean effect [19, 20]. This effect occurs in a single baseline OSA evaluation [19, 20]. The results of this study may apply for other Asian countries.

## 5. Conclusion

Transoral RFA may be effective in mild to moderate OSA as a first-line treatment with a low rate of complications. Baseline AHI is associated with preferable outcome by RFA.

## Figures and Tables

**Table 1:** Outcomes of radiofrequency ablation treatment in obstructive sleep apnea patients.

Baseline	Pretreatment	Posttreatment	*p* value
Snoring score	7.45 (2.04)	3.45 (1.75)	<0.001
Epworth sleepiness scale	10.14 (6.24)	5.51 (3.57)	<0.001
Apnea-hypopnea index	15.57 (7.33)	7.94 (5.69)	<0.001
Minimal saturation	79.90 (4.51)	85.10 (3.93)	<0.001

**Table 2:** Clinical features of obstructive sleep apnea patients who underwent radiofrequency therapy categorized by the posttreatment apnea hypopnea index (AHI).

Variables	AHI ≥ 5 *N* = 35	AHI < 5 *N* = 16	*p* value
Age, years	52 (21–64)	49 (31–64)	0.155
Male gender	27 (77.14%)	11 (68.75%)	0.281
BMI, kg/m^2^	25.60 (22.5–30)	25.20 (22.50–29.00)	0.503
Snoring score, before	8 (2–10)	8 (5–10)	0.812
Snoring score, after	3 (1–8)	3 (1–7)	0.785
ESS, before	9 (1–24)	11 (1–21)	0.692
ESS, after	5 (0–14)	5.5 (0–14)	0.691
AHI, before	17 (7–29)	8 (5–25)	<0.001
AHI, after	8 (5–23)	2.5 (0–4)	<0.001
Minimal saturation, before	82 (74–87)	75 (72–86)	<0.001
Minimal saturation, after	86 (80–95)	82.50 (76–89)	0.003
Pain score, after	0 (0–5)	0.5 (0–4)	0.303

Note: data presented as median (range) or numbers (percentage).

**Table 3:** Factors associated with having an apnea-hypopnea index (AHI) less than 5 times/hour after radiofrequency treatment in obstructive sleep apnea patients.

Variables	Odds ratio	Adjusted OR
Apnea-hypopnea index	0.830 (0.737, 0.934)	0.804 (0.699, 0.924)
Age	0.957 (0.894, 1.024)	0.952 (0.867, 1.047)
Male gender	0.652 (0.174, 2.438)	0.891 (0.167, 4.744)
Snoring score	0.995 (0.743, 1.333)	1.103 (0.734, 1.659)
Epworth sleepiness scale	1.009 (0.917, 1.110)	1.119 (0.960, 1.303)
Body mass index	0.898 (0.677, 1.191)	0.990 (0.699, 1.401)

Note: data presented as odds ratio (95% confidence interval); OR: odds ratio; all data were at baseline.

## References

[1] Lam J. C. M., Sharma S. K., Lam B. (2010). Obstructive sleep apnoea: definitions, epidemiology & natural history. *Indian Journal of Medical Research*.

[2] Takama N., Kurabayashi M. (2009). Influence of untreated sleep-disordered breathing on the long-term prognosis of patients with cardiovascular disease. *American Journal of Cardiology*.

[3] Epstein L. J., Kristo D., Strollo P. J. (2009). Clinical guideline for the evaluation, management and long-term care of obstructive sleep apnea in adults. *Journal of Clinical Sleep Medicine*.

[4] Martínez-García M.-Á., Capote F., Campos-Rodríguez F. (2013). Effect of CPAP on blood pressure in patients with obstructive sleep apnea and resistant hypertension: the HIPARCO randomized clinical trial. *Journal of the American Medical Association*.

[5] Pedrosa R. P., Drager L. F., de Paula L. K. G., Amaro A. C. S., Bortolotto L. A., Lorenzi-Filho G. (2013). Effects of OSA treatment on BP in patients with resistant hypertension: a randomized trial. *Chest*.

[6] Bradley T. D., Floras J. S. (2009). Obstructive sleep apnoea and its cardiovascular consequences. *The Lancet*.

[7] Powell N. B., Riley R. W., Troell R. J., Li K., Blumen M. B., Guilleminault C. (1998). Radiofrequency volumetric tissue reduction of the palate in subjects with sleep-disordered breathing. *Chest*.

[8] Aurora R. N., Casey K. R., Kristo D. (2010). Practice parameters for the surgical modifications of the upper airway for obstructive sleep apnea in adults. *Sleep*.

[9] Mirrakhimov A. E., Sooronbaev T., Mirrakhimov E. M. (2013). Prevalence of obstructive sleep apnea in Asian adults: a systematic review of the literature. *BMC Pulmonary Medicine*.

[10] Lam B., Lam D. C. L., Ip M. S. M. (2007). Obstructive sleep apnoea in Asia. *International Journal of Tuberculosis and Lung Disease*.

[11] Sher A. E., Flexon P. B., Hillman D. (2001). Temperature-controlled radiofrequency tissue volume reduction in the human soft palate. *Otolaryngology—Head and Neck Surgery*.

[12] Riley R. W., Powell N. B., Li K. K., Weaver E. M., Guilleminault C. (2003). An adjunctive method of radiofrequency volumetric tissue reduction of the tongue for OSAS. *Otolaryngology—Head and Neck Surgery*.

[13] Stuck B. A., Maurer J. T., Verse T., Hörmann K. (2002). Tongue base reduction with temperature-controlled radiofrequency volumetric tissue reduction for treatment of obstructive sleep apnea syndrome. *Acta Oto-Laryngologica*.

[14] Boudewyns A., Van de Heyning P. (2000). Temperature-controlled radiofrequency tissue volume reduction of the soft palate (Somnoplasty) in the treatment of habitual snoring: results of a European multicenter trial. *Acta Oto-Laryngologica*.

[15] Coleman S. C., Smith T. L. (2000). Midline radiofrequency tissue reduction of the palate for bothersome snoring and sleep-disordered breathing: a clinical trial. *Otolaryngology—Head and Neck Surgery*.

[16] Steward D. L., Weaver E. M., Woodson B. T. (2005). Multilevel temperature-controlled radiofrequency for obstructive sleep apnea: extended follow-up. *Otolaryngology—Head and Neck Surgery*.

[17] Li K. K., Powell N. B., Riley R. W., Troell R. J., Guilleminault C. (2000). Radiofrequency volumetric reduction of the palate: an extended follow-up study. *Otolaryngology—Head and Neck Surgery*.

[18] Iseri M., Balcioglu O. (2005). Radiofrequency versus injection snoreplasty in simple snoring. *Otolaryngology—Head and Neck Surgery*.

[19] Barnett A. G., van der Pols J. C., Dobson A. J. (2005). Regression to the mean: what it is and how to deal with it. *International Journal of Epidemiology*.

[20] Araghi M. H., Chen Y., Jagielski A. (2013). Effectiveness of lifestyle interventions on obstructive sleep apnea (OSA): systematic review and meta-analysis. *Sleep*.

